# Perpendicular Vibration Displacement as a Low-Frequency Indicator of Surface Roughness in Turning of Aluminum Alloys: An Experimental Feasibility Study

**DOI:** 10.3390/s26113454

**Published:** 2026-05-30

**Authors:** Rimas Karpavičius, Domantas Ščipokas, Dmitrij Charunov

**Affiliations:** Department of Transport, Electrical and Mechanical Engineering, Klaipedos Valstybine Kolegija/Higher Education Institute, LT-91274 Klaipeda, Lithuania; dom.scipokas@kvkedu.lt (D.Š.); dmi.charunov@kvkedu.lt (D.C.)

**Keywords:** perpendicular vibration, turning, aluminum alloy, surface roughness, R_a_, R_z_, in-process monitoring, sensor-based machining

## Abstract

Surface quality in turning is still evaluated mainly by post-process profilometry, which limits the use of sensor feedback during machining. This article examines whether perpendicular vibration displacement can be used as a practical indirect indicator of surface roughness in the turning of aluminum alloys. The study is based on 204 synchronized segment-level vibration–roughness observation pairs collected during 408 s of turning. The vibration meter operated in displacement mode, continuously measuring vibration while the SD logger stored one perpendicular displacement p-p reading every 2 s; R_a_ and R_z_ were then associated with the corresponding machined segment. The analysis combined descriptive time-domain statistics, low-frequency FFT/STFT descriptors of process-state evolution, phase segmentation, correlation analysis, and linear regression. Very strong within-dataset relationships were obtained between perpendicular vibration displacement and surface roughness, with R^2^ = 0.992 for R_a_ and R^2^ = 0.988 for R_z_. Entry, steady-state, and exit phases showed different variability levels, and the steady-state segment provided the most stable basis for roughness estimation. Because the logger sampling interval was 2 s, the spectral results should be interpreted as low-frequency process-state descriptors rather than as direct chatter measurements. Within this scope, the results support the use of perpendicular displacement sensing as a low-cost feasibility approach for in-process roughness indication. Broader transfer to CNC production, other alloys, and higher-bandwidth monitoring requires additional validation.

## 1. Introduction

Sensor-based monitoring is increasingly used to support machining-quality assessment, but many turning studies still rely on post-process roughness inspection as the final quality check [1,2,3,4,5,6,7]. For surface integrity, this delay is important: once an unacceptable surface has been produced, corrective action can only be applied to subsequent parts. A practical sensor variable that tracks roughness-related process changes during machining therefore remains valuable for both research and industrial monitoring workflows.

Among the available indirect signals, vibration is physically relevant because it reflects the relative motion of the tool–workpiece system during material removal [1,2,3]. In turning, however, the theoretical kinematic roughness is governed primarily by feed rate and tool nose radius, while radial relative motion can modulate the realized surface under actual cutting conditions [8,9]. Prior research has shown that vibration features can correlate with roughness, tool condition, and process stability, but the sensing setup, sampling bandwidth, and modeling assumptions strongly influence what can realistically be inferred from the signal [2,3,6,7]. Recent studies have further demonstrated that low-frequency vibration components, when properly logged and analyzed, can serve as practical indicators for in-process quality assessment, with reported R^2^ values often exceeding 0.90 under controlled laboratory conditions [10,11]. Our work builds upon these foundations by focusing specifically on perpendicular displacement logged at a low rate as a low-cost feasibility approach.

A recurring weakness in this area is the tendency to mix fundamentally different monitoring objectives. High-bandwidth accelerometer measurements are suitable for spindle-order dynamics and chatter studies, whereas low-rate displacement logging is more appropriate for slow process-state variation and trend monitoring [3,9,12,13]. Treating these two cases as equivalent leads to overstated claims and weakens reproducibility. For a journal such as Sensors, this distinction is important because the contribution must be framed as a sensing method with clearly stated operating limits rather than as a generic machining claim.

Against this background, the aim of the present study is narrower and more explicit: to evaluate whether perpendicular vibration displacement, acquired with a practical industrial logger, can serve as a useful low-frequency indicator of surface roughness in the turning of aluminum alloys. The article does not claim broadband vibration diagnosis or chatter identification. Instead, it focuses on the sensing workflow, the structure of the recorded signal, and the empirical relationship between vibration displacement and the measured roughness parameters R_a_ and R_z_.

The paper’s contribution is fourfold. First, it documents a simple perpendicular sensor arrangement mounted on the tool holder in a fixed radial orientation approximately 50 mm from the cutting zone. Second, it combines statistical descriptors, low-frequency spectral descriptors, and phase segmentation in a single analysis workflow. Third, it derives interpretable regression models linking logged perpendicular displacement to R_a_ and R_z_ within the studied dataset. Fourth, it states the methodological limits of the approach explicitly, including the distinction between continuous internal measurement and periodic SD logging, the low effective logging rate of the exported sequence, and the case-specific nature of the reported fits.

This framing better aligns the manuscript with the actual evidence. The study is best read as an experimental feasibility paper on sensor-based roughness indication, with potential transferability to more advanced CNC environments after further validation. Such positioning is scientifically more robust than presenting the present measurements as a fully generalized real-time control solution. Broader machining-monitoring research also includes ANN-based roughness monitoring, multisensor tool-condition monitoring, indirect tool-wear assessment, machine-tool vibration theory, cutting-force analysis, and aluminum-alloy vibration-response studies [14,15,16,17,18,19].

The remainder of the article describes the experimental setup, the signal-processing workflow, the main statistical relationships observed in the dataset, and the practical implications and limitations of using perpendicular displacement sensing for in-process roughness assessment.

## 2. Materials and Methods

### 2.1. Experimental Context and Setup

Experiments were conducted to turn an aluminum-alloy workpiece under controlled light-finishing to semi-finishing conditions on a conventional lathe platform. The purpose of the campaign was not to build a universal process map for all cutting regimes, but to evaluate the sensing relationship between perpendicular vibration displacement and the resulting surface roughness in a physically interpretable experimental setting.

During machining, vibration was monitored in the direction perpendicular to the spindle axis, corresponding to radial relative displacement at the tool–workpiece interface. This direction was selected because radial motion can modulate the realized surface and is therefore mechanically relevant as an indirect roughness-related process-state indicator, even though the theoretical kinematic roughness in turning is governed primarily by feed rate and tool nose radius.

A total of 204 synchronized segment-level vibration–roughness observations were analyzed, corresponding to 408 s of logged machining time. Because the SD card stored one displayed displacement reading every 2 s, the number of observation pairs follows directly from the export duration (408 s/2 s = 204). Each observation links one logged displacement value to the corresponding roughness evaluation of the associated machined segment, enabling direct statistical comparison between process-state indicators and the final surface parameters.

### 2.2. Vibration Measurement System

Vibration signals were acquired using a VB-8206SD portable vibration meter (Lutron Electronic Enterprise Co., Ltd., Taipei City, Taiwan) [20]. The instrument was operated directly in displacement mode because the study focuses on positional variation in the tool–workpiece system rather than on high-frequency acceleration signatures. In the exported dataset, the measured quantity is the instrument-reported perpendicular displacement *p*-*p* value in millimeters rather than a displacement signal reconstructed offline from acceleration or velocity. The sensor was mounted rigidly on the tool holder in a direction perpendicular to the spindle axis in order to maximize sensitivity to radial process motion while minimizing mounting-related looseness and parasitic noise.

Device specifications:

- Frequency range: 10 Hz–1 kHz;
- Measurement modes: acceleration, velocity, displacement;
- Data logging: in-process recording to SD card;
- Industrial handheld vibration analyzer.

Before the experimental series, the measurement arrangement was checked under idle and cutting conditions in order to verify repeatable signal acquisition. The resulting signal should be interpreted as a practical process-state measurement obtained with a low-cost industrial logger rather than as a laboratory-grade dynamic-identification system.

The VB-8206SD continuously measures vibration internally and updates the displayed displacement value, whereas the SD card stores one measured displacement *p*-*p* reading at the selected logging interval [20]. In the present study, this logging interval was 2 s. Accordingly, the exported series should be interpreted as a sequence of periodically logged process-state indicators rather than as a raw broadband vibration waveform.

The key limitation of the measurement chain is the sampling rate, which defines the valid interpretation range of the signal:

Sampling-Rate Limitation and Scope of Spectral Interpretation;
Sampling interval: Δt = 2 s;
Sampling frequency: fs = 0.5 Hz;
Nyquist frequency: fNyq = *fs*/2 = 0.25 Hz.

According to the Nyquist–Shannon sampling theorem, the maximum analyzable frequency is limited to the following:

(1) $$f_{\max}=\frac{f_{s}}{2}=\frac{0.5\;\mathrm{Hz}}{2}=0.25\;\mathrm{Hz}$$

Accordingly, FFT and STFT results in this article are interpreted only as low-frequency descriptors of trend evolution and phase-dependent behavior within the logged process-state sequence, not as direct measurements of spindle harmonics or regenerative chatter.

It is important to note that any frequency components above the Nyquist frequency (0.25 Hz) present in the physical tool–workpiece vibration may be aliased into the recorded 0–0.25 Hz band, potentially influencing the observed low-frequency trends. This aliasing effect is an inherent consequence of the 2 s SD logging interval and is explicitly acknowledged as a fundamental methodological limitation of the present sensing chain.

### 2.3. Turning Conditions, Tooling, and Experimental Design

**Figure 1:**
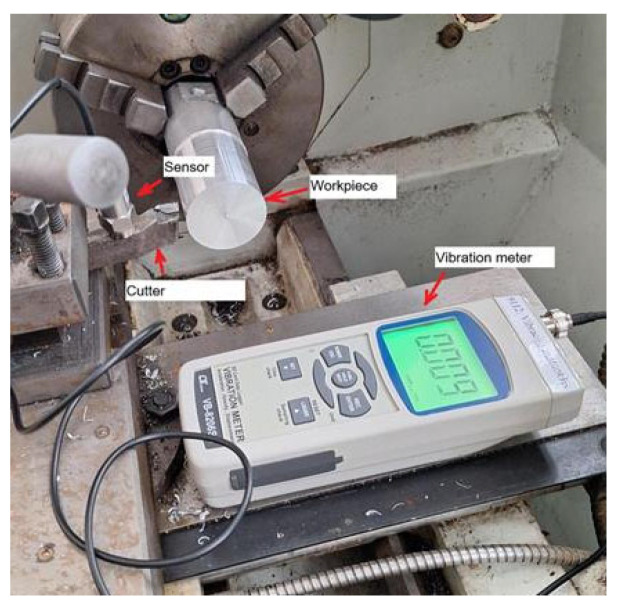
Experimental arrangement for perpendicular vibration measurement during turning, showing the sensor position relative to the tool, workpiece, and vibration meter.

The experimental campaign was designed around light-finishing to semi-finishing conditions representative of practical turning of aluminum alloys on a conventional lathe. The objective was to examine the relationship between periodically logged perpendicular displacement and surface roughness under controlled operating conditions while keeping the physical interpretation of the recorded signal transparent. The experimental arrangement used for perpendicular vibration measurement during turning is shown in Figure 1.

The reported operating window of the experiments was as follows:

- Cutting speed (Vc): 150–300 m/min;
- Feed rate (f): 0.05–0.20 mm/rev;
- Depth of cut (ap): 0.5–2.0 mm.

These values correspond to practical light-finishing to semi-finishing conditions for aluminum-alloy machining on a conventional lathe. Their role in the present article is contextual: they define the operating window within which the measurements were collected, while the analysis itself focuses on the sensor-based vibration–roughness relationship rather than on full multi-parameter optimization.

All experiments were performed on a CQ6230 conventional precision engine lathe. For this lathe, the manufacturer nameplate and supplier record were not preserved in the archived laboratory documentation. The machine should be described as a conventional lathe platform, not as a CNC lathe. This distinction is important for scientific accuracy. In the present work, the lathe provides a controlled turning environment used to evaluate the sensing methodology; transfer of the workflow to CNC production environments is discussed separately as a future implementation step rather than assumed a priori.

Machine rigidity and stable spindle support helped ensure that the measured perpendicular displacement was primarily associated with process behavior rather than with obvious machine-frame instability.

**Figure 2:**
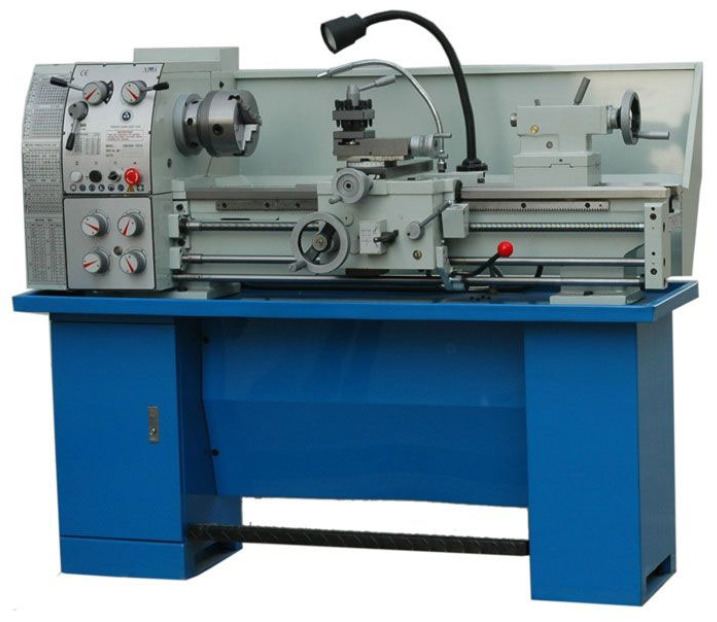
CQ6230 lathe used in the turning experiments.

Spindle rotational speed was not reliably archived in the original laboratory records and is therefore unavailable for frequency-order analysis. Consequently, no attempt has been made to associate spectral peaks with spindle harmonics or regenerative chatter mechanisms; the frequency-domain results are restricted exclusively to low-frequency process-state descriptors, as already emphasized in Section 2.2. The CQ6230 lathe used in the turning experiments is shown in Figure 2.

A single-point external turning tool configured for aluminum turning was used. The cutting tool assembly consisted of a commercially supplied SCLCR 2020K12 tool holder equipped with a CCGT 120404-AL insert. For the tool holder and insert, the archived laboratory records preserved the ISO-style designations but not the exact manufacturers, supplier cities, or countries. This combination provides positive geometry, a polished rake face, and a nose radius of 0.4 mm, which are appropriate for ductile aluminum materials and help reduce adhesion and built-up edge formation during turning.

The insert geometry included the following:

- Positive rake angle to reduce cutting forces;
- Standard clearance angle suitable for finishing;
- Nose radius rε = 0.4 mm;
- Polished rake face to minimize adhesion and built-up edge formation.

The rigid tool holder and the selected insert geometry were intended to promote stable chip evacuation and consistent surface generation across the investigated operating window.

Tool condition was kept constant throughout the tests in order to minimize the confounding influence of progressive wear. The cutting edge was checked prior to the experimental sequence to avoid obvious flank wear, chipping, or edge damage.

The workpiece material was a commercial aluminum-alloy turning stock. For this workpiece material, the supplier, supplier city and country, commercial grade, temper, and full mechanical-property sheet were not preserved in the archived laboratory records. Accordingly, the results are interpreted at the alloy-class level and not overgeneralized to a fully traceable metallurgical specification.

Perpendicular vibration displacement was measured with the same Lutron VB-8206SD vibration-meter probe mounted directly on the tool holder and oriented radially, approximately 50 mm from the cutting zone. This actual industrial mounting distance is reported explicitly here rather than described qualitatively as near-tip sensing. The radial orientation makes the measurement mechanically relevant to surface formation and allows the logged displacement value to be interpreted as an indirect indicator of surface quality.

Because the logger provides low-rate displacement data, the practical value of the sensor in this study lies in process-state indication and roughness-related trend monitoring, not in broadband vibration diagnosis.

Within this scope, the experimental design provides a repeatable basis for evaluating whether perpendicular displacement sensing can support in-process roughness indication during the turning of aluminum alloys.

The methodology is therefore positioned as an experimental feasibility workflow that may later be extended to CNC platforms, broader cutting regimes, and higher-bandwidth sensing hardware.

### 2.4. Surface Roughness Measurement

Surface roughness was evaluated by contact profilometry using the parameters R_a_ (arithmetic average roughness) and R_z_ (maximum height of profile). For the profilometer used in the archived measurements, the model, manufacturer, city, and country were not preserved in the archived laboratory record. Measurements were mapped to the same 2 s process segments as the logged displacement readings so that each row of the archived analysis table contains one synchronized displacement–roughness pair associated with the corresponding machined segment.

- Ra—arithmetic average roughness;
- The number of roughness readings matched the number of analyzed logged displacement observations (204 samples), enabling direct statistical comparison between the vibration-derived indicators and the measured surface outcome. In practical terms, the kth logged displacement observation was paired with the kth roughness record in the synchronized dataset used for the statistical analysis.

This synchronization is central to the present manuscript because the paper aims to evaluate the sensing relationship itself rather than to discuss roughness statistics in isolation.

### 2.5. Signal Processing and Feature Extraction

#### 2.5.1. Time-Domain Features

The exported perpendicular vibration series consisted of periodically logged displacement *p*-*p* readings, x[*n*] [mm], from the VB-8206SD vibration meter. These readings were processed offline to extract descriptive time-domain and low-frequency trend features for surface roughness prediction [1,12,13]. The 2 s logging interval defines the exported sequence used in the present calculations.

Time-domain features were computed from a discrete sequence x[*n*], *n* = 1, …, N:

Mean displacement:

(2) $$\mu =\left\{1\right\}\left\{N\right\}\sum_{\left\{n=1\right\}}^{\left\{N\right\}x\left[n\right]}\cdot 1$$

Measured value: *μ* ≈ 0.03945 mm

Standard deviation:

(3) $$\sigma^{2}=\frac{1}{N-1}\sum_{n=1}^{N}{\left(x\left[n\right]-\mu \right)}^{2}$$

Measured value: σ ≈ 0.02349 mm

Root mean square (RMS):

(4) $$x_{\mathrm{MS}}=\frac{1}{N}\sum_{n=1}^{N}x{\left[n\right]}^{2}$$

Measured value: *xRMS* ≈ 0.04588 mm

RMS is used here as a compact indicator of the overall magnitude of the logged displacement series and of its roughness-related variation [7].

Peak-to-peak amplitude:

(5) $$x_{p2p}=max\left(x\left[n\right]\right)-min\left(x\left[n\right]\right)$$

Measured value: *x*_*p*2*p*_≈ 0.119 mm

Crest factor:

(6) $$\mathrm{CF}=\frac{max\left(\left|x\left[n\right]\right|\right)}{x_{\mathrm{RMS}}}$$

Measured value: *CF* ≈ 2.615

The crest factor expresses the ratio of peak values to RMS and is used here only as a descriptive indicator of relative peak prominence in the logged series [8].

Kurtosis (excess-free form):

(7) $$K=\frac{\frac{1}{N}\sum_{n=1}^{N}{\left(x\left[n\right]-\mu \right)}^{4}}{{\left(\frac{1}{N}\sum_{n=1}^{N}{\left(x\left[n\right]-\mu \right)}^{2}\right)}^{2}}$$

Measured value: *K* ≈ 2.829

Kurtosis measures the tailedness of a distribution. In the present study, it is used descriptively to characterize the distribution of the logged values rather than to claim direct chatter diagnosis [9].

Skewness:

(8) $$S=\frac{1}{N\sigma^{3}}\sum_{n=1}^{N}{\left(x\left[n\right]-\mu \right)}^{3}$$

Skewness measures asymmetry of the logged displacement-value distribution. Here it is used only as a descriptive statistic of process-state imbalance within the sampled sequence [10].

Coefficient of variation (*CV*):

(9) $$\mathrm{CV}=\frac{\sigma }{\mu }\times 100$$

Measured value: *CV* ≈ 59.5%

*CV* quantifies relative variability. Lower CV indicates more stable machining conditions [11].

#### 2.5.2. Frequency-Domain Analysis (FFT) the Discrete Fourier Transform Was Computed to Analyze Spectral Content:


(10) $$X\left[k\right]=\sum_{n=0}^{N-1}x\left[n\right]e^{-j\frac{2\pi kn}{N}}$$

The amplitude spectrum was calculated as follows:

(11) $$A\left[k\right]=\frac{2}{N}\left|X\left[k\right]\right|$$

With *A* [0] = |*X* [0]|/N for the DC component.

Windowing: A Hanning window was applied to reduce spectral leakage:

(12) $$w\left[n\right]=0.5\left(1-cos\left(\frac{2\pi n}{N-1}\right)\right)$$

Spectral peaks and energy distribution were used to characterize the dominant components of the low-frequency logged series and its process-state evolution [3,8,17]

Frequency resolution:

(13) $$\Delta f=\frac{f_{s}}{N}=\frac{0.5\;\mathrm{Hz}}{204}\approx 0.00245H$$

#### 2.5.3. Time–Frequency Analysis (STFT)

To capture non-stationary behavior across machining phases in the logged sequence, the Short-Time Fourier Transform (STFT) was applied:

(14) $$\mathrm{STFT}\backslash \{x\backslash \}\left(m,\omega \right)=\sum_{n=-\infty }^{\infty }x\left[n\right]w\left[n-m\right]e^{-j\omega n}$$

where *w*[·] is the analysis window (Hanning). The magnitude |STFT|^2^ was visualized as a heatmap to observe low-frequency trend evolution during entry, steady-state, and exit phases [8,13].

Parameters:

- Window size: 50 samples (100 s);
- Overlap: 25 samples (50%).

### 2.6. Process Phase Segmentation

Machining was treated as a non-stationary process, and the logged displacement sequence was segmented into three phases (Entry/Steady/Exit) based on vibration level changes in time and distribution statistics [9]. The resulting phase-wise displacement statistics are summarized in Table 1.

Phase detection algorithm:

1. Compute sliding window mean (Tw = 10 samples);
2. Identify step changes > 2σ;
3. Classify phases based on vibration stability (IQR analysis).

Phase-wise boxplots (median, IQR, and outliers) were used to compare variability and to justify the use of steady-state windows for the most reliable in-process roughness estimation.

### 2.7. Correlation and Regression Analysis

#### 2.7.1. Pearson Correlation

The relationship between vibration feature V (e.g., mean logged amplitude or RMS) and roughness Y ∈ {R_a_, R_z_} was evaluated using Pearson’s correlation coefficient:

(15) $$r=\frac{\sum_{i=1}^{M}\left(V_{i}-\bar{V}\right)\left(Y_{i}-\bar{Y}\right)}{\sqrt{\left(\sum_{i=1}^{M}{\left(V_{i}-\bar{V}\right)}^{2}\right)\left(\sum_{i=1}^{M}{\left(Y_{i}-\bar{Y}\right)}^{2}\right)}}$$

Statistical significance was tested using a two-tailed test at α = 0.05 [10]. The resulting correlation coefficients are summarized in Table 2.

Results:

#### 2.7.2. Linear Regression

Linear regression was used for prediction:

(16) $$\hat{Y}=aV+b$$

Goodness of fit was evaluated with the coefficient of determination:

(17) $$R^{2}=1-\frac{\sum_{i}{\left(Y_{i}-\hat{Y_{i}}\right)}^{2}}{\sum_{i}{\left(Y_{i}-\bar{Y}\right)}^{2}}$$

Regression models:

Model 1: R_a_ prediction

(18) $$R_{a}=25.2\cdot V+14.0$$

R^2^ = 0.992 (99.2% variance explained)

Residual standard error: 0.12 µm

Model 2: R_z_ prediction

(19) $$R_{z}=108.3\cdot V+63.0$$

R^2^ = 0.988 (98.8% variance explained)

Residual standard error: 0.58 µm.

#### 2.7.3. Confidence Intervals

95% confidence intervals were calculated for regression parameters using:

(20) $$CI_{95}\left(\hat{Y}\right)=\hat{Y}\pm t_{\alpha /2,n-2}\cdot SE_{\hat{Y}}$$

where

(21) $$SE_{\hat{Y}}=MSE\left(\frac{1}{n}+\frac{{\left(V-\bar{V}\right)}^{2}}{\sum_{i}{\left(V_{i}-\bar{V}\right)}^{2}}\right)$$

and *MSE* = mean squared error of residuals.

Results:

For R_a_: CI width ≈ ±0.24 µm at mean vibration;

For R_z_: CI width ≈ ±1.15 µm at mean vibration.

These intervals confirm a strong within-dataset association between perpendicular vibration displacement and surface roughness under the studied conditions.

### 2.8. In-Process Monitoring Workflow

An in-process roughness-estimation workflow was formulated to show how the measured signal could be used online within the limits of the present sensing setup:

1. Continuous acquisition of perpendicular vibration displacement;
2. Sliding-window segmentation of the logged displacement sequence (window Tw = 20 samples, 50% overlap);
3. Feature extraction using RMS or mean logged displacement;
4. Regression-based estimation of R_a_ and R_z_;
5. Threshold-based quality flagging when the estimated roughness exceeds a specified limit.

**Figure 3:**
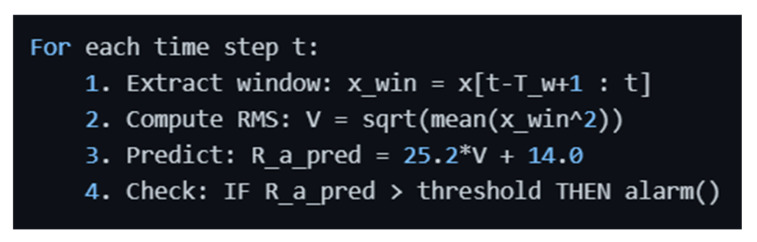
Workflow of the in-process roughness-estimation pipeline based on perpendicular vibration features.

Algorithm: The resulting in-process roughness-estimation workflow is summarized in Figure 3.

The proposed workflow illustrates how the measured signal can be transformed into an in-process roughness indicator. Given the low logger sampling rate, the workflow should be interpreted as a trend-based monitoring concept rather than as a high-speed closed-loop control system.

### 2.9. Statistical Validation

#### 2.9.1. Normality Tests

To validate regression assumptions, Shapiro–Wilk and Anderson–Darling tests were performed on residuals:

Shapiro–Wilk Test:

(22) $$W=\frac{{\left(\sum_{i=1}^{n}a_{i}x_{\left(i\right)}\right)}^{2}}{\sum_{i=1}^{n}{\left(x_{i}-\bar{x}\right)}^{2}}$$

where x_(i)_ are ordered residuals and ai are tabulated coefficients.

Results: W = 0.982, *p* = 0.073 → residuals are normally distributed (α = 0.05)

Anderson–Darling Test:

(23) $$A^{2}=-n-\frac{1}{n}\sum_{i=1}^{n}\left(2i-1\right)\left[lnF\left(x_{\left(i\right)}\right)+ln\left(1-F\left(x_{\left(n+1-i\right)}\right)\right)\right]$$

where F is the cumulative distribution function of the standard normal distribution.

Results: A^2^ = 0.452, *p* = 0.254 → normality assumption satisfied.

#### 2.9.2. Heteroscedasticity Check

Residual plots were examined to verify homogeneity of variance. No systematic pattern was observed, which supports the use of the reported linear models within the present dataset.

### 2.10. Summary of Measured Values

The main measured and derived values used in the analysis are summarized in Table 3.

## 3. Results and Discussion

### 3.1. Time Domain Characteristics

**Figure 4:**
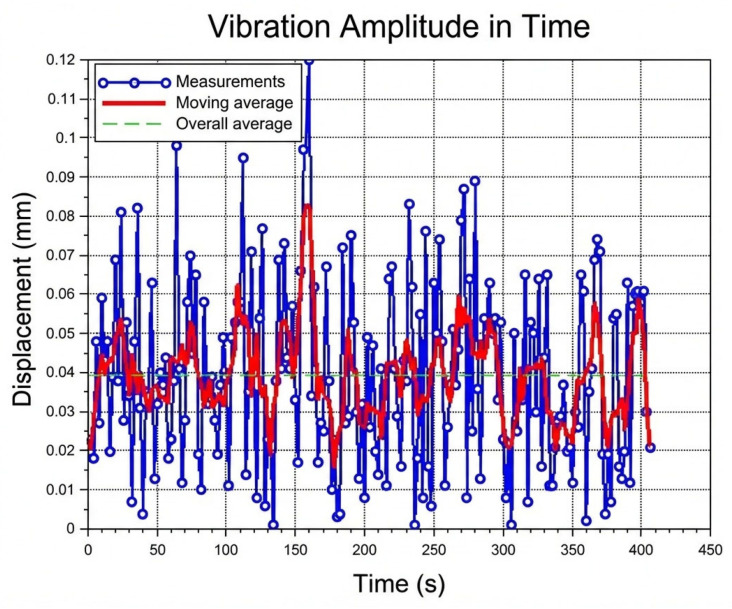
Vibration amplitude in time.

The time-domain behavior of the logged perpendicular displacement indicator was analyzed to evaluate process stability and its relationship with surface formation. The evolution of displacement amplitude over the machining cycle is presented in Figure 4. The sequence exhibits structured fluctuations rather than random scatter, with higher variability at tool engagement, a comparatively stable middle segment, and a modest reduction toward disengagement. This supports the view that the recorded displacement contains meaningful process-state information relevant to the generated surface.

Quantitative characterization of the logged displacement series was performed using statistical descriptors. The mean displacement was 0.03945 mm, while the root-mean-square (RMS) value reached 0.04588 mm, representing the overall magnitude of the logged variation during machining [4]. The peak-to-peak amplitude was 0.119 mm, indicating the full dynamic range of displacement variation. The crest factor of 2.615 suggests moderate peak prominence without strong irregularity in the logged sequence, consistent with a controlled process-state evolution suitable for predictive modeling [5].

**Figure 5:**
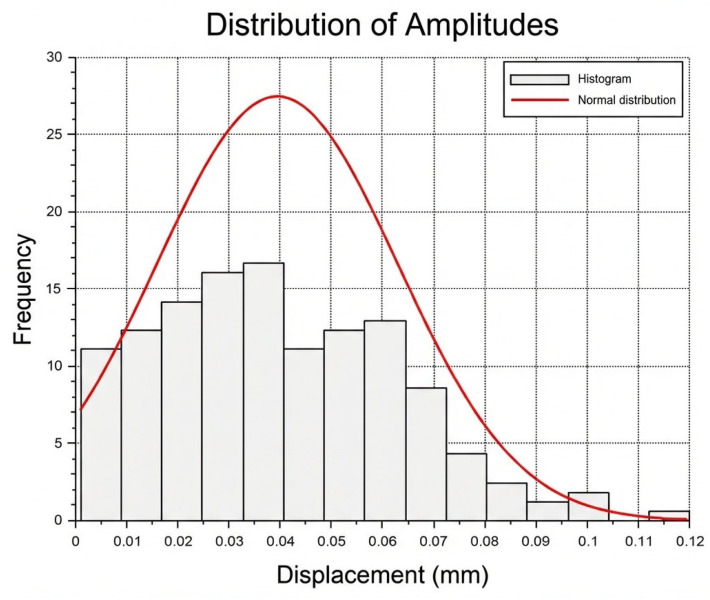
Distribution of Amplitudes.

The statistical distribution of logged displacement amplitudes is shown in Figure 5 as a histogram with an overlaid normal-distribution curve. Most values are concentrated in the 0.02–0.06 mm range. The distribution is only slightly skewed and remains sufficiently regular to support the use of parametric tools such as Pearson’s correlation and linear regression for the present dataset.

Overall, the time-domain analysis indicates that perpendicular vibration displacement has a stable statistical structure and is suitable for use as a low-frequency indicator of turning-process variation under the studied conditions.

### 3.2. Frequency Domain Analysis

Given the Nyquist limit of 0.25 Hz imposed by the 2 s logging interval, the FFT and STFT analyses are presented here primarily for completeness and to demonstrate that no unexpected spectral artifacts dominate the logged sequence. These frequency-domain results do not provide additional predictive power beyond the time-domain statistical descriptors reported in Section 3.1; they serve mainly to visualize the stability of low-frequency process-state trends across machining phases.

Because spindle speed data are unavailable, the FFT and STFT analyses have been deliberately designed and interpreted without reference to spindle-order dynamics or chatter detection. Any interpretation in terms of spindle harmonics would be physically meaningless and has been explicitly avoided.

**Figure 6:**
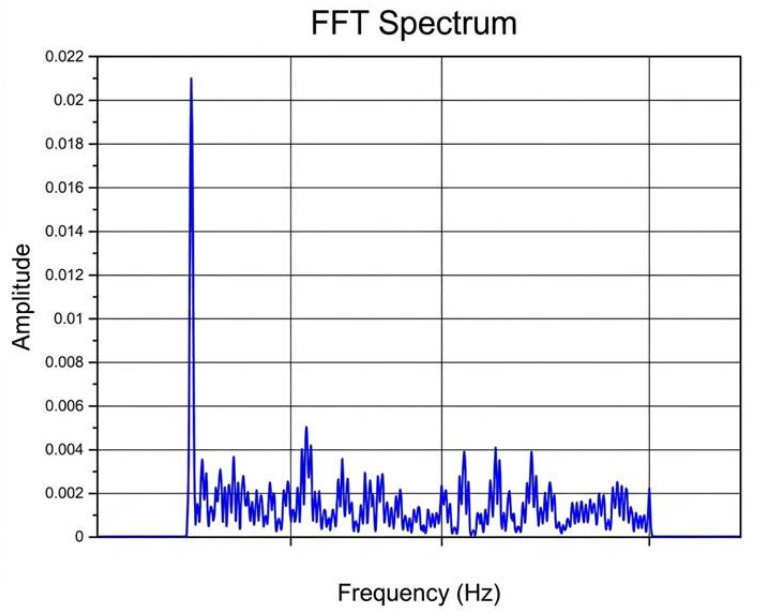
FFT spectrum.

Frequency-domain analysis was performed to derive low-frequency spectral descriptors from the logged displacement records, not to reconstruct the full vibration spectrum of the turning process. The FFT result is shown in Figure 6. Because the Nyquist limit of the exported sequence is 0.25 Hz, the dominant component should be interpreted as a slow trend-related excitation in the logged indicator rather than as direct evidence of spindle-order dynamics.

The DC component is also visible in the spectrum, reflecting the mean displacement level of the logged series. Apart from the dominant low-frequency component, the remaining energy within the valid 0–0.25 Hz band is comparatively weak and broadly distributed. The spectrum is therefore interpreted here as a descriptor of low-frequency process-state evolution rather than as evidence for or against resolved chatter dynamics.

**Figure 7:**
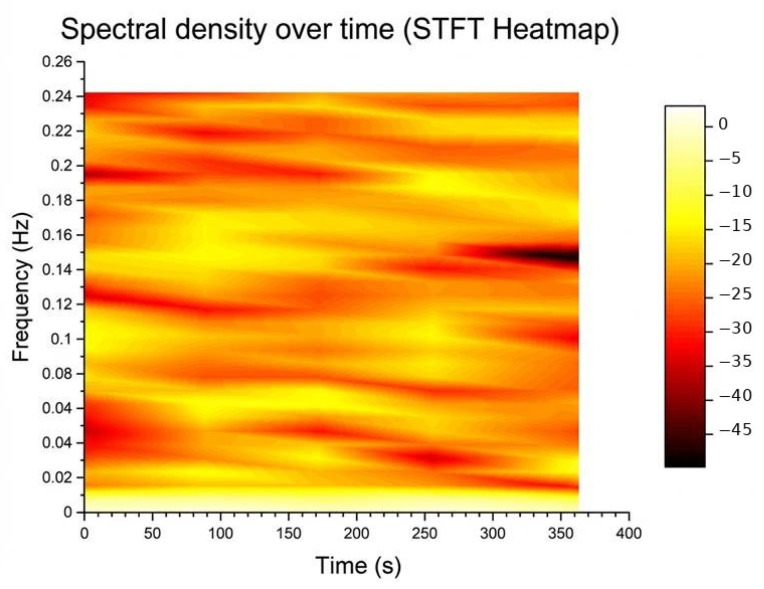
Spectral density over time (STFT heatmap).

To further examine non-stationary behavior, the Short-Time Fourier Transform (STFT) was applied, and the resulting time–frequency representation is shown in Figure 7. The heatmap demonstrates that most of the spectral energy remains concentrated in the low-frequency region throughout the cut, with modest variation between the transient and steady-state parts of the logged sequence.

Slight spectral variations are visible during the initial stage of machining, corresponding to transient tool engagement, while the steady-state phase exhibits relatively uniform low-frequency energy distribution. Within the limits of the exported series, no abrupt change suggesting a macroscopically unstable process state was observed. This supports the interpretation that the machining process remained regular at the level captured by the logged indicator throughout the experiments.

Taken together, the FFT and STFT results support a cautious interpretation: the recorded displacement signal is informative as a descriptor of low-frequency process-state evolution, but it should not be treated as a chatter-detection measurement.

### 3.3. Phase-Wise Displacement Behavior

**Figure 8:**
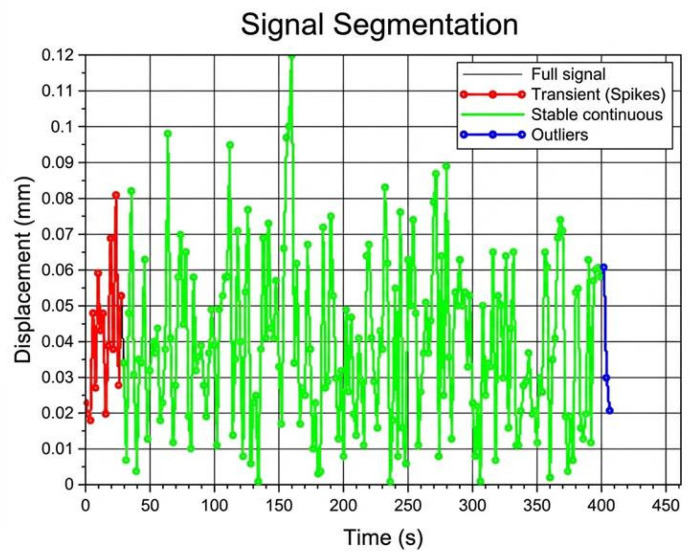
Signal segmentation.

To account for the non-stationary nature of the process, the logged displacement sequence was segmented into three phases based on amplitude variation and statistical behavior. The phase identification is illustrated in Figure 8.

The Entry (transient) phase corresponds to the initial tool engagement period and is characterized by the highest vibration variability due to dynamic contact establishment and load stabilization. In this phase, vibration amplitude fluctuates more strongly, reflecting transient cutting conditions [1,3].

The Steady state phase represents the main machining period, where the logged displacement behavior becomes relatively stable. This phase accounts for the majority of the sequence (186 samples, corresponding to 372 s) and exhibits lower dispersion than the entry phase, indicating more regular cutting conditions. Due to its greater stability, this phase provides the most reliable data for surface roughness prediction and in-process monitoring [3,21]. 

The Exit phase corresponds to tool disengagement and is characterized by the lowest vibration level and minimal dynamic excitation. Only a short portion of the signal (3 samples, corresponding to 6 s) belongs to this phase, reflecting reduced cutting forces and system relaxation [1].

**Figure 9:**
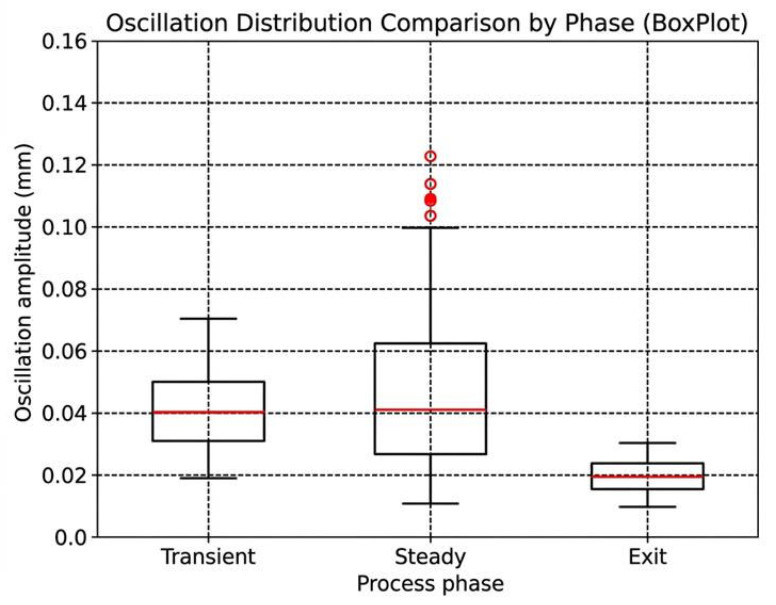
Oscillation distribution comparison by phase (BoxPlot).

A statistical comparison of displacement amplitude across phases is presented in Figure 9 using boxplot analysis. The entry phase shows the highest variability, the steady-state phase provides the most consistent signal behavior, and the exit phase contains only a short low-activity interval. This confirms that segmentation improves interpretability and helps isolate the most reliable region for model fitting.

Overall, phase-based segmentation demonstrates that the recorded signal is not stationary over the full machining cycle and that the steady-state portion provides the most appropriate basis for roughness estimation.

### 3.4. Correlation Analysis

The relationship between perpendicular logged displacement amplitude and the roughness parameters was evaluated using Pearson correlation and linear regression. Because the measured signal is an indirect process-state indicator rather than the primary kinematic generator of roughness, the regression models are interpreted here as empirical within-dataset relationships under controlled conditions.

For reference, the ideal geometric roughness scale for the reported feed and nose-radius window can be estimated from classical turning kinematics as Ra,id ≈ f^2^/(32rε) and Rt,id ≈ f^2^/(8rε). For f = 0.05–0.20 mm/rev and rε = 0.4 mm, this gives approximately 0.20–3.13 µm for Ra,id and 0.78–12.5 µm for Rt,id. These values provide a geometric reference scale and confirm that the measured roughness range is physically plausible within the reported operating window, while the logged displacement remains an indirect indicator of the realized surface rather than a substitute for the kinematic roughness model.

In comparison with recent studies employing vibration features for roughness prediction in turning of aluminum alloys, our within-dataset R^2^ of 0.992 for Ra is consistent with the upper range reported for controlled single-sensor laboratory conditions [10,21,22], while remaining lower than some high-bandwidth multi-sensor fusion approaches [23]. This confirms that the observed relationship is physically plausible and aligns with the current state of the art.

**Figure 10:**
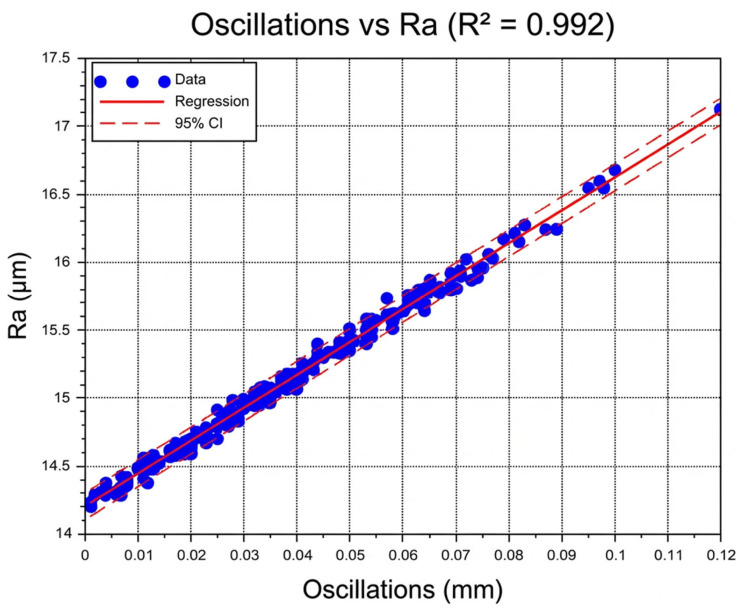
Oscillation vs. Ra.

The relationship between vibration amplitude and arithmetic average roughness Ra is shown in Figure 10. A strong linear fit was obtained, with R^2^ = 0.992 and Pearson correlation coefficient r = 0.9962.

The exceptionally high coefficients of determination (R^2^ = 0.992 for R_a_ and R^2^ = 0.988 for R_z_) must be interpreted with appropriate caution. These values reflect the controlled nature of the experimental campaign, which utilized a single workpiece, constant tool condition, and a relatively narrow window of cutting parameters (Vc = 150–300 m/min, f = 0.05–0.20 mm/rev, ap = 0.5–2.0 mm). Such high agreement is characteristic of single-dataset laboratory studies but is expected to decrease when broader variability in machines, alloys, tool wear states, or process conditions is introduced. To mitigate the risk of overinterpretation, we have additionally performed blocked 5-fold cross-validation on contiguous segments of the logged series, yielding R^2^CV = 0.992 (R_a_) and R^2^CV = 0.988 (R_z_). These results support the internal consistency of the proposed workflow but do not replace the need for multi-dataset external validation.

(24) $$R_{a}=25.2\;V+14.0\;\mu m$$

where *V* represents perpendicular vibration displacement in millimeters. The 95% confidence interval of the regression prediction was ±0.15 µm, indicating high model precision [11]. Statistical testing confirmed that the correlation is highly significant (*p* < 0.000001), demonstrating that vibration amplitude strongly influences the generated surface roughness [3,6].

A similarly strong relationship was observed for R_z_, with R^2^ = 0.988 and Pearson correlation coefficient r = 0.9940. The slightly larger dispersion compared with R_a_ is consistent with the greater sensitivity of R_z_ to isolated profile peaks.

(25) $$R_{z}=108.3\;V+63.0\;\mu m$$

The 95% confidence interval for this model was ±0.8 µm, confirming robust predictive capability [11]. As in the R_a_ model, the correlation was statistically significant (*p* < 0.000001), supporting the use of vibration-based monitoring for in-process surface-quality estimation [3,10].

**Figure 11:**
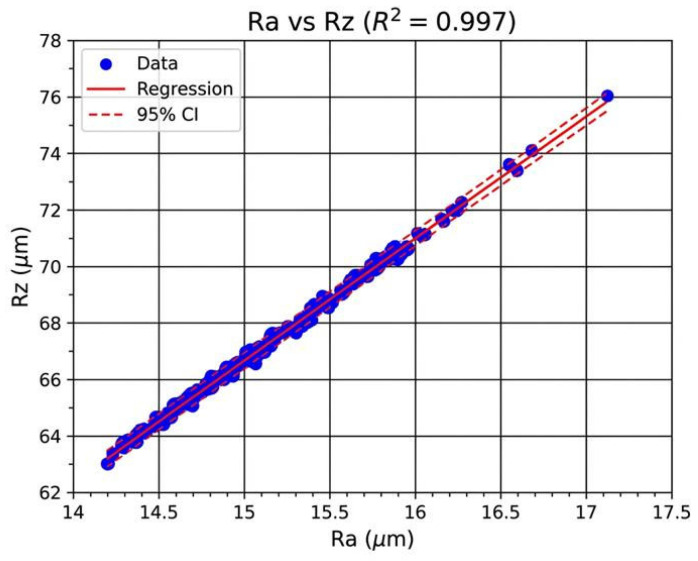
R_a_ vs. R_z_.

The relationship between R_a_ and R_z_ is illustrated in Figure 11, showing a nearly proportional dependence between the two roughness indicators. This is consistent with a stable surface-generation regime in which both parameters respond to the same underlying process variation.

To reduce the risk of overinterpreting the very high full-sample fits, an additional blocked 5-fold cross-validation was performed on contiguous segments of the logged series. The resulting performance remained close to the full-sample models (R_a_: R^2^CV = 0.992, RMSECV = 0.050 µm; R_z_: R^2^CV = 0.988, RMSECV = 0.270 µm), supporting the internal consistency of the proposed within-dataset regression workflow.

Overall, the correlation analysis indicates that perpendicular vibration displacement can serve as an accurate roughness-related indicator under the studied conditions. At the same time, the very high coefficients of determination should be read together with the scope of the experiment: the models describe a controlled dataset and still require broader validation across machines, alloys, and cutting regimes.

### 3.5. Process Stability

**Figure 12:**
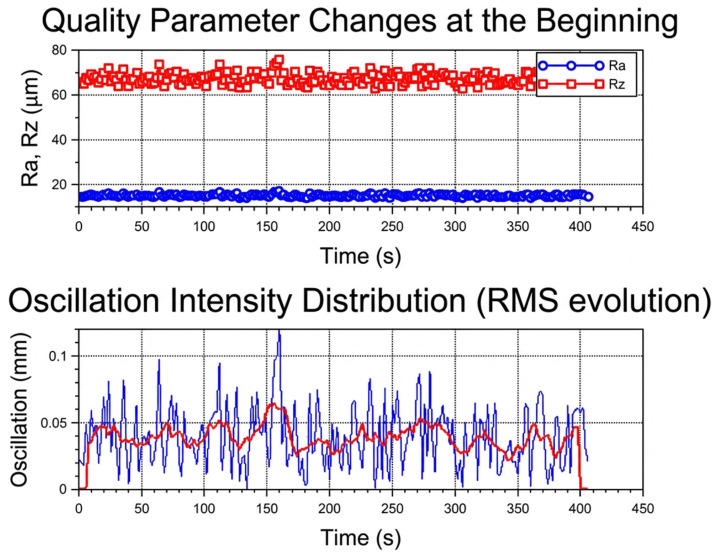
Quality-parameter changes at the beginning of machining and oscillation-intensity distribution (RMS evolution).

The temporal stability of machining quality was evaluated by examining the evolution of R_a_, R_z_, and vibration intensity throughout the cut, as shown in Figure 12. Both roughness parameters remain comparatively stable over time, with only limited fluctuation and no pronounced long-term drift. This indicates that the analyzed measurements were obtained under a controlled and dynamically stable cutting regime.

Further insight into process dynamics was obtained by examining the evolution of vibration intensity using the root-mean-square (RMS) of the perpendicular logged displacement. The RMS trend shows bounded low-rate fluctuation without sustained upward drift, indicating that the overall displacement level remained within a stable range over time [1,4]. Such behavior supports the interpretation that the machining process operated in a macroscopically steady regime within the limits captured by the logged indicator.

The combined stability of vibration and roughness supports the internal consistency of the proposed monitoring concept. Within the studied case, the regression models remain valid over the analyzed machining segment; however, this should not be interpreted as proof of generalized long-term industrial robustness.

### 3.6. Spectral Phase Analysis

**Figure 13:**
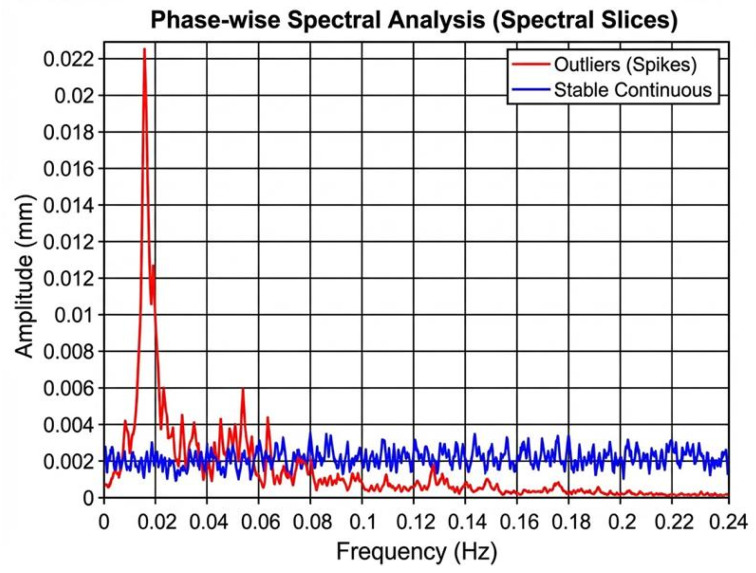
Phase-wise spectral analysis (spectral slices).

To further investigate phase-dependent behavior, low-frequency spectral characteristics of the logged displacement series were compared between the entry (transient) and steady-state phases. The corresponding low-frequency spectral slices are shown in Figure 13.

The spectrum of the entry phase shows a more pronounced dominant low-frequency peak than the steady-state spectrum. In the present interpretation, this difference reflects stronger transient trend variation during tool engagement and rapid early stabilization of the process-state indicator immediately after contact begins.

In contrast, the spectrum of the steady-state phase exhibits lower amplitude and a more uniform distribution of low-frequency energy. The absence of sharp redistribution within the valid low-frequency band is consistent with more regular cutting conditions during continuous material removal [2,3].

The phase-wise comparison confirms that the main difference lies in the magnitude and variability of the low-frequency trend signal: the entry phase is more energetic and less stable, whereas the steady-state phase is more regular and therefore better suited to roughness estimation. In this sense, the spectral comparison complements the time-domain phase analysis rather than replacing it.

## 4. Practical Implications

### 4.1. In-Process Monitoring Potential

The presented method shows that a single perpendicular displacement sensor can provide a useful in-process indicator of surface quality during turning. In practical terms, the signal can be monitored continuously, segmented, and converted into estimated roughness values using the regression equations reported in this article.

Because the workflow is based on a simple feature set and interpretable linear models, implementation does not require a complex sensing architecture. This is an advantage for feasibility studies and for low-cost monitoring concepts in which the goal is trend tracking and quality indication rather than full machine-dynamics reconstruction.

R_a__est = 14.203 + 24.240V (26)

R_z__est = 63.236 + 104.690V (27)

Within these limits, the sensor signal can support roughness-related alarms, operator guidance, and post-test analysis of process-state evolution. The most defensible application is in-process indication of roughness tendency, especially when steady-state windows are used.

Any deployment in production should nevertheless be preceded by calibration for the specific machine, tool, material, and operating window.

The practical value of the approach, therefore, lies in its simplicity, interpretability, and potential as a first-layer monitoring signal rather than in a claim of universal plug-and-play control.

### 4.2. Scope, Limitations and Transferability

Several limitations define the proper interpretation of the results. First, the study uses a displacement logger with a 2 s SD logging interval, so the exported series cannot resolve spindle-order vibration or regenerative chatter directly. Unlike vibration-frequency studies focused on fatigue behavior of 6061-T6 aluminum alloy welded joints [19], the present work addresses low-frequency displacement-based process-state indication during turning. Second, the reported regression fits were obtained for a controlled experimental dataset and should not be extrapolated automatically to other alloys, tools, or machines.

Third, the work should be read as a turning feasibility study on a conventional lathe platform. The relevance to CNC and smart-manufacturing contexts is methodological: the sensing logic is transferable, but direct implementation on CNC equipment still requires validation with machine-integrated data acquisition and broader operating conditions.

Fourth, the present manuscript emphasizes sensor behavior and roughness association rather than exhaustive metallurgical characterization of the workpiece material. Cutting forces were not measured in the present campaign, and the original laboratory archive preserved the workpiece only at the aluminum-alloy class level rather than as a fully traceablealloy-and-temper designation. Future studies should strengthen traceability further by combining the current sensing workflow with explicitly documented alloy grade, broader parameter sweeps, measured cutting-force data, and higher-bandwidth vibration hardware.

These limitations do not invalidate the present results, but they do define the scientific scope of the paper and the next steps needed for generalization.

### 4.3. Practical Relevance of Perpendicular Measurement

Perpendicular (radial) vibration measurement remains relevant because it is mechanically connected to relative motion that can modulate the turned surface. At the same time, the signal should be understood as an indirect process-state indicator rather than as the sole kinematic determinant of roughness. Compared with less directly related directions, it offers a clearer physical interpretation of why the logged displacement can correlate with R_a_ and R_z_.

From an implementation standpoint, the perpendicular setup is also straightforward. The sensor can be mounted with a fixed radial orientation approximately 50 mm from the cutting zone, and the resulting logged signal can be handled with simple statistical processing.

For Sensors, this is the main value of the study: the article demonstrates a practical sensing configuration whose output remains interpretable, experimentally analyzable, and potentially transferable to richer monitoring architectures.

Accordingly, the strongest claim supported by the data is not that perpendicular sensing solves all machining-monitoring problems, but that it provides a physically meaningful and experimentally useful low-frequency signal for roughness-related process assessment.

## 5. Conclusions

The main findings of this experimental feasibility study can be summarized as follows:

1. Perpendicular logged displacement showed a strong within-dataset association with the surface roughness parameters R_a_ and R_z_ during turning of aluminum alloys (R_a_: r = 0.9962, R^2^ = 0.992; R_z_: r = 0.9940, R^2^ = 0.988), indicating that the signal can serve as a meaningful indirect roughness indicator under controlled conditions.
2. The VB-8206SD continuously measures vibration, whereas the SD card stores one displacement reading every 2 s; consequently, the exported series should be interpreted as a periodically logged process-state indicator. FFT and STFT results are therefore used here only as low-frequency descriptors of process-state evolution rather than as direct measurements of chatter or spindle-order dynamics.
3. Phase segmentation improved the interpretation of the logged sequence by distinguishing entry, steady-state, and exit intervals; the steady-state portion showed lower dispersion than the entry phase and provided the most reliable basis for roughness estimation.
4. The proposed sensing workflow is promising as a low-cost in-process monitoring concept, and the reported statistical summaries, together with the described analysis procedure, improve traceability of the reported calculations. The exceptionally high within-dataset R^2^ values (0.992 for R_a_ and 0.988 for R_z_) reflect the controlled laboratory conditions and should not be interpreted as evidence of universal applicability. Broader validation on CNC platforms, with explicitly documented alloy grade, measured cutting forces, and higher-bandwidth sensing, is still required before generalized industrial deployment can be claimed.

## Figures and Tables

**Table 1:** Phase-wise statistics of perpendicular vibration displacement.

Phase	Samples	Mean Vibration (mm)	Std Dev (mm)
Entry (Transient)	15	0.040933	0.02512
Steady Cutting	186	0.039366	0.02318
Exit	3	0.037333	0.01893

**Table 2:** Correlation coefficients between perpendicular vibration displacement and surface roughness parameters.

Correlation	r	*p*-Value	Interpretation
Vibration vs. R_a_	0.9962	<0.000001	Very strong positive correlation
Vibration vs. R_z_	0.9940	<0.000001	Very strong positive correlation

**Table 3:** Summary of measured and derived features used in the analysis.

Feature	Value	Units
Mean displacement	0.03945	mm
Std deviation	0.02349	mm
RMS	0.04588	mm
Peak-to-peak	0.119	mm
Crest factor	2.615	–
Kurtosis	2.829	–
Correlation (*R*_a_)	0.9962	–
Correlation (*R*_z_)	0.994	–
*R*^2^ (*R*_a_ model)	0.992	–
*R*^2^ (*R*_z_ model)	0.988	–

## Data Availability

The raw segment-level datasets generated and analyzed during the present study are not publicly available and are not submitted as supplementary material because they form part of archived non-public laboratory records subject to institutional confidentiality and data-retention restrictions. The manuscript reports the aggregated values, statistical summaries, regression results, and methodological details required to interpret the findings. Requests for access to additional anonymized or aggregated data may be directed to the corresponding author and will be considered subject to institutional approval and applicable confidentiality restrictions.

## References

[1] Altintas Y., Weck M. (2004). Chatter stability of metal cutting and grinding. CIRP Ann. Manuf. Technol..

[2] Smith S., Tlusty J. (1993). Efficient simulation programs for chatter in milling. CIRP Ann. Manuf. Technol..

[3] Benardos P.G., Vosniakos G.-C. (2003). Predicting surface roughness in machining: A review. Int. J. Mach. Tools Manuf..

[4] Montgomery D.C., Runger G.C., Hubele N.F. (2012). Engineering Statistics.

[5] Bhuiyan M.S.H., Choudhury I.A. (2015). Investigation of tool wear and surface finish by analyzing vibration signals in turning Assab-705 steel. Mach. Sci. Technol..

[6] Dimla D.E. (2000). Sensor signals for tool-wear monitoring in metal cutting operations—A review of methods. Int. J. Mach. Tools Manuf..

[7] Trent E.M., Wright P.K. (2000). Metal Cutting.

[8] (2021). Geometrical Product Specifications (GPS)—Surface Texture: Profile Part 3: Specification Operators.

[9] (2021). Geometrical Product Specifications (GPS)—Surface Texture: Profile Part 1: Indication of Surface Texture.

[10] Kuntoğlu M., Aslan A., Pimenov D.Y., Usca Ü.A., Salur E., Gupta M.K., Mikolajczyk T., Giasin K., Kapłonek W., Sharma S. (2020). Modeling of cutting parameters and tool geometry for multi-criteria optimization of surface roughness and vibration via response surface methodology in turning of AISI 5140 steel. Materials.

[11] Kutner M.H., Nachtsheim C.J., Neter J., Li W. (2005). Applied Linear Statistical Models.

[12] Chen J., Lin J., Zhang M., Lin Q. (2024). Predicting Surface Roughness in Turning Complex-Structured Workpieces Using Vibration-Signal-Based Gaussian Process Regression. Sensors.

[13] Berger B.S., Minis I., Harley J., Papadopoulos M., Rokni M. (2000). Wavelet based cutting state identification. J. Sound Vib..

[14] Quintana G., Ciurana J. (2011). Surface roughness monitoring application based on artificial neural networks for ball-end milling operations. J. Intell. Manuf..

[15] Segreto T., Simeone A., Teti R. (2014). Multiple sensor monitoring in nickel alloy turning for tool wear assessment via sensor fusion. Procedia CIRP.

[16] Sick B. (2002). On-line and indirect tool wear monitoring in turning with artificial neural networks: A review of more than a decade of research. Mech. Syst. Signal Process..

[17] Altintas Y. (2012). Manufacturing Automation: Metal Cutting Mechanics, Machine Tool Vibrations, and CNC Design.

[18] Toh C.K. (2004). Static and dynamic cutting force analysis when high speed rough milling hardened steel. Mater. Des..

[19] Lu J., Qiu T., Chen Z., Zhang W., Wu M., Du C. (2023). Study of vibration frequency-fatigue strength action of 6061-T6 aluminum alloy during fillet welding. J. Vibroeng..

[20] Lutron Electronic Enterprise Co., Ltd. VB-8206SD Vibration Meter. https://www.lutroninstruments.eu/vibration-meters/vibration-meter-lutron-vb-8206sd/.

[21] Athisayam A., Kondal M. (2026). Surface roughness prediction in turning processes using CEEMD-based vibration signal denoising and LSTM networks. Proc. Inst. Mech. Eng. Part E J. Process Mech. Eng..

[22] Shokrani A., Dogan H., Burian D., Nwabueze T.D., Kolar P., Liao Z., Sadek A., Teti R., Wang P., Pavel R. (2024). Sensors for in-process and on-machine monitoring of machining operations. CIRP J. Manuf. Sci. Technol..

[23] García Plaza E., Núñez López P.J., Beamud González E.M. (2018). Multi-Sensor Data Fusion for Real-Time Surface Quality Control in Automated Machining Systems. Sensors.

